# Black Bean (*Phaseolus vulgaris* L.) Polyphenolic Extract Exerts Antioxidant and Antiaging Potential

**DOI:** 10.3390/molecules26216716

**Published:** 2021-11-06

**Authors:** David Fonseca-Hernández, Eugenia Del Carmen Lugo-Cervantes, Antonio Escobedo-Reyes, Luis Mojica

**Affiliations:** Tecnología Alimentaria, Centro de Investigación y Asistencia en Tecnología y Diseño del Estado de Jalisco, A.C. CIATEJ, Unidad Zapopan, Camino Arenero 1227, El Bajío, Zapopan 45019, Jalisco, Mexico; dafonseca_al@ciatej.edu.mx (D.F.-H.); elugo@ciatej.mx (E.D.C.L.-C.); aescobedo@ciatej.mx (A.E.-R.)

**Keywords:** phenolic compounds, black bean, tyrosinase, elastase, antioxidant, supercritical fluids extraction

## Abstract

Phenolic compounds present in common beans (*Phaseolus vulgaris* L.) have been reported to possess antimicrobial, anti-inflammatory and ultraviolet radiation (UVR) protective properties. UVR from sunlight, which consists of UV-B and UV-A radiations, induces reactive oxygen species (ROS) and free radical formation, consequently activating proteinases and enzymes such as elastase and tyrosinase, leading to premature skin aging. The objective of this work was to extract, characterize and evaluate the antioxidant and antiaging potential of polyphenols from a black bean endemic variety. The polyphenolic extract was obtained from black beans by supercritical fluid extraction (SFE) using CO_2_ with a mixture of water–ethanol as a cosolvent and conventional leaching with a mixture of water–ethanol as solvent. The polyphenolic extracts were purified and characterized, and antioxidant potential, tyrosinase and elastase inhibitory potentials were measured. The extract obtained using the SFE method using CO_2_ and H_2_O–Ethanol (50:50 *v*/*v*) as a cosolvent showed the highest total phenolic compounds yield, with 66.60 ± 7.41 mg GAE/g coat (*p* > 0.05) and 7.30 ± 0.64 mg C3GE/g coat (*p* < 0.05) of anthocyanins compared to conventional leaching. Nineteen tentative phenolic compounds were identified in leaching crude extract using ESI-QTOF. Quercetin-3-D-galactoside was identified in crude and purified extracts. The purified SFC extract showed IC_50_ 0.05 ± 0.002 and IC_50_ 0.21 ± 0.008 mg/mL for DPPH and ABTS, respectively. The lowest IC_50_ value of tyrosinase inhibition was 0.143 ± 0.02 mg/mL and 0.005 ± 0.003 mg/mL of elastase inhibition for leaching purified extract. Phenolic compounds presented theoretical free energy values ranging from −5.3 to −7.8 kcal/mol for tyrosinase and −2.5 to −6.8 kcal/mol for elastase in molecular docking (in silico) studies. The results suggest that the purified extracts obtained by SFE or conventional leaching extraction could act as antioxidant and antiaging ingredients for cosmeceutical applications.

## 1. Introduction

Skin aging is a degradative process with morphological and functional changes, such as elasticity, strength loss and hyperpigmentation. Intrinsic and extrinsic factors are the leading causes of these degradative changes [1]. Intrinsic factors can be defined as physiological changes regulated by inherited genes and the passing of time. On the other hand, extrinsic factors are associated with environmental factors, including sunlight exposure, harmful chemicals and air pollution. The combination of these factors leads to premature skin aging [2]. The sun’s ultraviolet radiation (UVR) consists of two types of radiation: UV-B (290–320 nm) and UV-A (320–400 nm), which induces oxidative stress in the skin by increasing reactive oxygen species and free radical production [3]. Therefore, this leads to the activation of specific metabolic pathways involved in regulating and expressing proteinases that degrade structural proteins present in the extracellular matrix (ECM) [4]. Elastases are serine proteases related to ECM degradation that are released during inflammation by neutrophils and dermal fibroblasts. Elastase cleaves elastin, a structural protein that provides elasticity to the skin [5]. UVR can also lead to excessive melanin production by tyrosinase, originating skin hyperpigmentation and aging spots. Therefore, diverse tyrosinase inhibitors can be used as skin-whitening agents [6]. Several secondary metabolites from plants are used as active ingredients in cosmetic formulations because they modulate the enzymes involved in the skin-aging process [7].

Common bean (*Phaseolus vulgaris* L.) is a source of phenolic compounds found in seed coats. Phenolic compounds are secondary metabolites produced constitutively in the plants and have an important role in protecting them from herbivores and microbial infections. They also serve as attractants for pollinators, UV protectants and signal molecules in the formation of root nodules for nitrogen-fixing in legumes [8]. The most common phenolic compounds found in common beans are p-coumaric acid, ferulic acid, myricetin, kaempferol and anthocyanins such as delphinidin, malvidin, cyanidin and pelargonidin [9]. Polyphenols from dietary sources and plants exhibit diverse beneficial biological activities, such as antimicrobial, anti-inflammatory, anticarcinogenic and anti-aging properties. Reports have indicated that phenolic compounds could protect the skin from UVR effects when ingested or applied topically [10]. Supercritical fluids extraction (SFE) is a non-conventional procedure to obtain bioactive compounds from natural sources. It offers advantages in contrast with conventional extraction procedures such as leaching (LC) and Soxhlet extraction. SFE reduces environmental impact and minimizes energy costs. Therefore, its use is important for different industries [11]. On the other hand, conventional leaching is a solid-extraction technique that can be performed with low-cost equipment and can be adapted to extract various specific compounds by using different solvents, temperatures and agitation combinations. The main disadvantages of this method are the long extraction times and the large volumes of solvent used [12]. The objective of this work was to extract, characterize and evaluate the antioxidant and antiaging potentials of polyphenols from an endemic variety of black bean.

## 2. Results

### 2.1. Conventional Leaching and SFE Extraction

The extract obtained using the SFE method with H_2_O–Ethanol 50% as a cosolvent showed the highest yield of total phenolic compounds, with 66.60 ± 7.41 mg GAE/g coat and 7.30 ± 0.64 mg C3GE/g coat of anthocyanins (Table 1). The conventional LC process using H_2_O–Ethanol 50% as a solvent presented 59.83 ± 4.86 mg GAE/g coat of total phenolic compounds and anthocyanins with a concentration of 5.87 ± 0.21 mg C3GE/g coat, presenting no statistical difference (*p* > 0.05) for phenolic compounds.

### 2.2. Tentative Dentification of Phenolic Compounds by ESI-QTOF

Nineteen tentative phenolic compounds were identified in the LC and SFE for crude and purified extracts. The main tentative anthocyanins identified were malvidin-3-glucoside, cyanidin-3-glucoside, delphinidin-3-glucoside and petunidin-3-O-β glucoside. These compounds are present in both crude and purified conventional LC extracts. However, cyanidin-3-glucoside was found in SFE crude and purified extracts only (Table 2). The non-colored phenolic compounds identified in both crude and purified extracts were quercetin-3-D-galactoside, naringenin, catechin, myricetin, gallic, ferulic and rosmarinic acid. However, other non-colored phenolic compounds were identified among the extracts, such as genistein, rutin, glycitein, daidzein and phenolic acids such as sinapic, a protocatechuic, caffeic and p-coumaric acid.

### 2.3. Antioxidant Capacity

**Figure 1:**
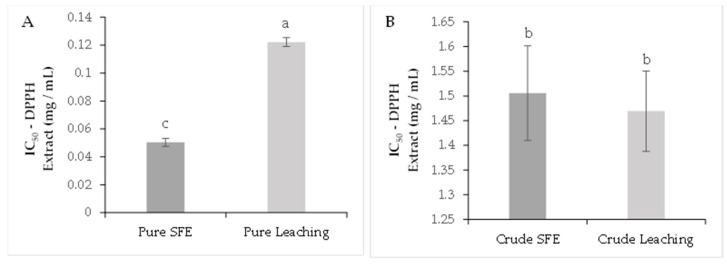
DPPH antioxidant capacity analysis. Purified extracts (**A**), crude extracts (**B**). The results are shown as mean ± standard error. Different letters indicate significant differences among treatments (*p* < 0.05) (Tukey’s HSD post hoc analysis).

**Figure 2:**
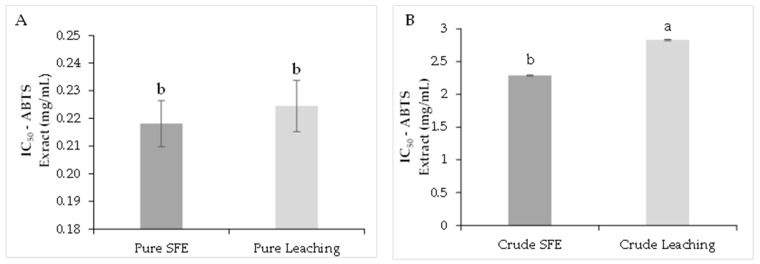
ABTS antioxidant capacity analysis. Purified extracts (**A**), crude extracts (**B**). The results are shown as mean ± standard error. Different letters indicate significant differences among treatments (*p* < 0.05) (Tukey’s HSD post hoc analysis).

As shown in Figure 1A, the purified extract obtained by SFE presented an IC_50_ value of 0.05 ± 0.002 mg/mL compared to the LC extract with a value of 0.12 ± 0.003 mg/mL with significant differences (*p* < 0.05) for DPPH scavenging assay. This indicates that the purification process enhances the antioxidant capacity of the extract, and it can be compared with the lower values registered for the crude extracts (Figure 1B). For the ABTS assay, the SFE-purified extract registered an IC_50_ of 0.21 ± 0.008 mg/mL and 0.22 ± 0.009 mg/mL, with no significant differences (*p* > 0.05) for the LC-purified extract (Figure 2A). On the other hand, the IC_50_ for the crude extracts were 2.28 ± 0.09 mg/mL and 2.82 ± 0.14 mg/mL for SFC and LC, respectively (Figure 2B).

### 2.4. Tyrosinase Inhibitory Potential

**Figure 3:**
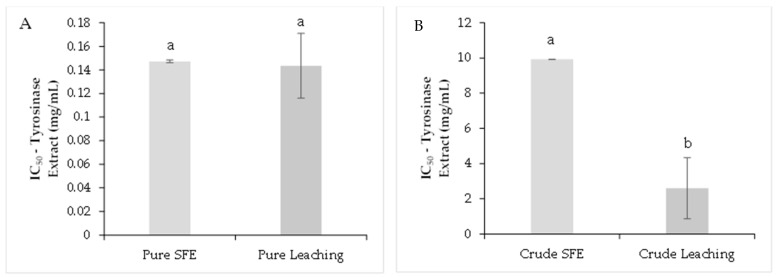
Tyrosinase inhibition capacity analysis. Purified extracts (**A**), crude extracts (**B**). The results are shown as mean ± standard error. Different letters indicate significant differences among treatments (*p* < 0.05) (Tukey’s HSD post hoc analysis).

The cosmetic industry is interested in using active ingredients that can reduce hyperpigmentation on the skin. This effect can be achieved by inhibiting or reducing the activity of the tyrosinase enzyme. The purified extracts obtained by SFE and LC present IC_50_ for tyrosinase with values of 0.147 ± 0.02 mg/mL and 0.143 ± 0.02 mg/mL, respectively (Figure 3A). In the raw extracts, the values were 9.92 ± 1.73 mg/mL for SFE and 2.59 ± 0.22 mg/mL for LC (Figure 3B). As positive control, the kojic acid showed an IC_50_ of 0.012 ± 0.001 mg/mL; this compound is used as a whitening agent in cosmetic products.

### 2.5. Elastase Inhibitory Potential

**Figure 4:**
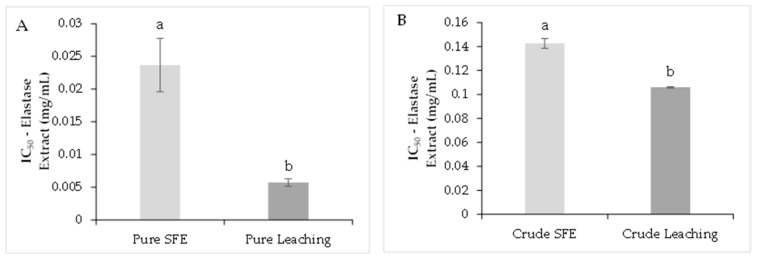
Elastase inhibition capacity analysis. Purified extracts (**A**), crude extracts (**B**). The results are shown as mean ± standard error. Different letters indicate significant differences among treatments (*p* < 0.05) (Tukey’s HSD post hoc analysis).

The purified and crude extracts recovered by LC showed a higher capacity to inhibit elastase activity than SFE (Figure 4A,B). The SFE-purified extract showed an IC_50_ of 0.023 ± 0.07 mg/mL, and for the purified LC extract, the IC_50_ was significantly lower (*p* < 0.05) with a value of 0.005 ± 0.01 mg/mL. In the case of crude extracts, SFE presented 0.142 ± 0.01 mg/mL and 0.105 mg/mL for the LC extract (*p* < 0.05). The catechin was used as a positive control, presenting an IC_50_ of 5.33 ± 0.07 mg/L.

### 2.6. Molecular Docking (In Silico Assay)

Molecular docking analysis was performed to predict the interactions between the tentative phenolic compounds present in the extract and the interaction with tyrosinase and elastase enzymes. The phenolic compounds studied presented theoretical free energy values ranging from −5.3 to −7.8 kcal/mol for tyrosinase. In the case of elastase, theoretical binding affinities ranged from −2.4 to −6.8 kcal/mol. Kojic acid and catechin were also evaluated as positive controls, presenting free energies of −5.5 and −6.9 for tyrosinase and elastase, respectively (Table 3).

## 3. Discussion

Based on the results obtained from the extraction process, there were no statistical differences (*p* > 0.05) between total phenolics and anthocyanins content in SFE extraction with H_2_O and H_2_O–ethanol as cosolvents. Nevertheless, the highest content of these compounds was found in the extraction with H_2_O–ethanol (50:50 *v/v*) for SFE and LC processes. In a similar study, the authors compared alternative and conventional extraction processes with a black bean variety [13]. The extraction using SFE obtained 11.09 mg GAE/g bean coat of total phenolic compounds and a total of 2.64 mg C3GE/g bean coat, demonstrating that this extraction method with the same cosolvents is better compared to the conventional extraction process. During SFE extraction, the cosolvent interacts directly with the bean coat without the interference of the cotyledon. This saves time by eliminating the manual dehulling process [13]. The use of hydroethanolic solutions over pure solvents for the phenolic compound extraction is recommended due to the high solubility of the compounds in the solvent, allowing a higher release from the material matrix [14]. Black beans present a high anthocyanins concentration, and their content differs between varieties [9]. Endemic common bean varieties from Chiapas, Mexico, contain a high content of these secondary metabolites. In a previous study, common bean anthocyanins were extracted using conventional leaching, with values ranging from 5.30 ± 0.09 to 9.42 ± 0.03 mg C3GE/g seed coat for black bean varieties [15]. The anthocyanin concentration obtained from our endemic black bean is similar to these values. Black bean seed coats are removed from cotyledons before the conventional leaching extraction process. The cotyledon contains proteins and complex carbohydrates that could be used to develop functional ingredients such as protein concentrates or carbohydrate extracts [16,17].

ESI-QTOF by direct infusion offers a rapid mass spectral analysis while analyzing samples at atmospheric pressure, making it a suitable technique for studying secondary metabolites in plant materials and food [18]. A large variety of tentative phenolic compounds in common beans were identified in the crude LC extract. However, five tentative phenolic compounds were not identified in the LC purified extract. Caffeic acid, p-coumaric acid, sinapic acid, daidzin and genistein were not present in the purified extract. Catechin is the main phenolic compound in black-colored beans and is mainly found in the coat [19]. Cyanidin-3-glucoside has been reported in endemic black bean varieties (*Phaseolus vulgaris* L.) from Chiapas [17]. This metabolite offers a beneficial effect by protecting the skin from the adverse effects of UVB radiation through the modulation of signaling pathways involved in skin photoaging, such as the MAP kinase pathway [20]. The phenolic compounds identified in this black bean cultivar have been previously reported in other common bean cultivars. Ferulic acid, p-coumaric acid, caffeic acid and sinapic acid were identified in 16 common bean varieties (*Phaseolus vulgaris* L.). Caffeic and ferulic acids are phenolic acids of great interest for the cosmetic industry and were found in black bean varieties [21]. Catechin, ferulic acid, gallic acid, rutin, myricetin and p-coumaric acid were identified in commercial black bean extracts obtained through supercritical fluid extraction, pressurized liquid extraction and conventional leaching [13]. It has been reported that there are three important anthocyanins in a Mexican black bean cultivar: delphinidin, malvidin and petunidin glucosides, among other phenolics [22]. Other non-colored phenolic compounds such as genistein, glycitein, daidzin and rosmarinic acid are found in the coat of diverse common beans, making this legume an important source of these secondary metabolites [23].

Dietary anthocyanins are important for the food industry as natural colorants. They are obtained from edible fruits such as blueberries, raspberries, blackberries, purple sweet potatoes, black currants and red cabbages. For the extraction of anthocyanins from these materials, the whole fruit is used. Black beans contain high amounts of anthocyanin in their coats; the cotyledons could be used to obtain value-added ingredients such as protein concentrates or isolates and complex carbohydrate extracts, with potential applications in the food industry [16,17].

XAD-7HP polymeric resin is widely used for the purification of anthocyanin-rich extracts. The adsorption capacity of the resins depends on the pore size and surface area. Also, the interaction between hydrogen groups of phenolic compounds and polarity groups of the XAD-7HP resin is important. However, other resins could be used to purify these compounds to minimize the loss of specific phenolics [24]. On the other hand, phenolic compounds recovered by SFE might be susceptible to degradation due to temperature and pressure factors during the extraction [13].

The results obtained for antioxidant capacity show that the purification process enhances the antioxidant potential of the extract by concentrating the compounds. Phenolic compounds can scavenge free radicals efficiently due to the catechol group present in the chemical structure. The polyphenols transfer hydrogen atoms easily because of the low dissociation energies present in the -OH group [13,23]. Similarly, plant extracts with the potential to be used as cosmeceutical ingredients, such as *Artocarpus species* extracts, present a high antioxidant potential for ABTS (IC_50_ < 0.03 mg/mL). These results show that black bean-purified extracts can scavenge cationic radicals [25]. Oxidative stress is related to extracellular matrix alterations in the dermis, causing premature skin aging. The production of high concentrations of radical oxygen species mediates the activation of receptor tyrosine kinases (RTKs) and downstream signaling pathways, which are associated with the expression of matrix metalloproteinases [26]. These endopeptidases are related to the degradation of structural proteins in the dermis, such as collagen and elastin [27]. In the cosmetic industry, antioxidants have two purposes: they can act as active ingredients to protect the skin from oxidative stress or stabilize other ingredients present in the cosmetic formulation [28]. The purified LC extracts could be used as antioxidant ingredients for the development of cosmeceuticals.

There is an interest in using active ingredients that can reduce hyperpigmentation in the skin. This could be achieved by inhibiting or reducing the activity of the tyrosinase enzyme [29]. LC and SFE extracts presented no statistical differences (*p* > 0.05) by reducing the enzymatic activity of tyrosinase. However, kojic acid showed the lowest IC_50_ of 0.012 ± 0.001 mg/mL. This compound is used as a whitening agent in cosmetic products. Nevertheless, the prolonged use of this compound increases skin sensibility and allergic reactions [30]. Flavonoids are natural tyrosinase inhibitors; gallic acid has significant inhibition capacity and is found in all black bean crude and purified extracts [31]. Shaving white (*Dendrobium* spp.) is an orchid from Thailand; its ethanolic extract can be used as a skin whitening agent. The extract has an IC_50_ value of 907.22 ± 14.32 µg/mL using L-DOPA as substrate [32]. The agricultural byproducts can be the source of bioactive compounds that can be used as whitening agents. Cocoa pod extract contains flavonoids that present an IC_50_ of 357.95 µg/mL for tyrosinase inhibition potential. In comparison, the LC-purified extract from black beans shows better anti-tyrosinase potential [33].

Elastin is an important structural protein in the ECM; elastase is a key enzyme related to the degradation of these proteins. Exposure to ultraviolet radiation in the skin increases this enzyme’s expression and activity, causing elastin degradation [34]. Grape pomace obtained from white wine production contains polyphenols with elastase inhibitory potential. A concentration of 35.3 µg/mL of the extract resulted in 73% inhibition of the enzyme. This extract contains polyphenols such as gallic acid, catechin and quercetin, found in the black bean extract [5]. On the other hand, herbal extracts from Asia, such as *Manilkara zapota* (L.), can be used as active ingredients for elastase inhibition. This plant is used as an herbal medicine due to its high content of antioxidants. The ethanolic extract shows an IC_50_ of 35.73 ± 0.61 µg/mL for inhibiting the elastase enzyme. In comparison, the SFE and the LC purified extract present a lower potential to block the enzyme [35].

**Figure 5:**
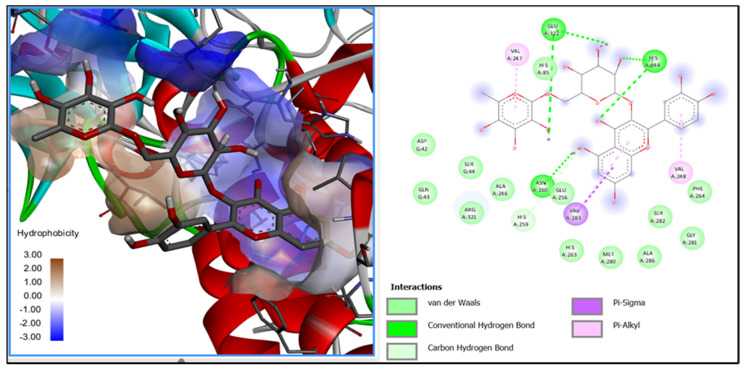
Molecular docking diagram for rutin interaction with the catalytic site of tyrosinase. Results represent the mean ± SD of at least two independent experiments.

According to the molecular docking analysis, rutin presented a predicted free energy value of −8.5 kcal/mol compared to kojic acid, with a theoretical value of −5.5 kcal/mol for the tyrosinase enzyme. Cyanidin, malvidin, petunidin 3-glucosides and quercetin 3-D galactoside showed low theoretical free energy values, ranging from −7.1 to −7.8 kcal/mol. The interaction of rutin with the enzyme’s active site was mainly through hydrogen bonds and π-sigma interactions (Figure 5). Anthocyanins have a high inhibitory capacity compared to other flavonoids such as kaempferol, quercetin 3-O-(6″-O-galloyl)-β-galactopyranoside or quercetin 3-O-β-galactopyranoside, which are found in medicinal plants [6]. For elastase analysis, catechin showed a predicted free energy value of −6.9 kcal/mol, followed by rosmarinic acid with −6.8 kcal/mol and naringenin with −6.7 kcal/mol. Catechin was used as a positive control, and it showed the best binding interactions with the active site of the enzyme; however, it is also present in the extracts. The main interactions with the amino acid residues of the active site are van der Waals and hydrogen bonds. Other compounds used as cosmetic ingredients, such as caffeine, presented a free energy value of −3.36 kcal/mol [36].

## 4. Materials and Methods

### 4.1. Materials

The “black bean” (*Phaseolus vulgaris* L.) was cultivated in November 2018 and collected in March 2019 in Chiapas, Mexico. The grains were stored at 4 °C until use. Methanol (≥98%), ethanol (≥99%), sodium carbonate, Folin–Ciocalteu 2 N reagent, gallic acid, chlorohydric acid, potassium chloride, sodium acetate, Amberlite^®^ XAD-7HP, formic acid, 2,2-diphenyl-1-picrylhydrazyl (DPPH), 2,2′-azino-bis(3-ethylbenzothiazoline)-6-sulfonic acid (ABTS), 6-hydroxy-2, 5, 7, 8-tetramethylchrome-2-carboxylic acid (Trolox^®^), potassium persulfate, methanol mass grade, sodium phosphate dibasic, monopotassium phosphate, sodium hydroxide, tris base, 3,4-Dihydroxy-L-phenylalanine (L-DOPA), kojic acid, N-succinyl-ala-ala-ala-p-nitroanilide (SANA), dimethyl sulfoxide (DMSO), citric acid, (±) catechin hydrate, tyrosinase from mushroom (EC 1.14.18.1) and elastase from porcine pancreas (EC 3.4.21.36), quercetin-3-D-galactoside (≥98%), delphinidin 3-O-glucoside (≥98%), malvidin 3-O-glucoside (≥98%) and cyanidin 3-O-glucoside (≥98%) were purchased from Sigma-Aldrich (St. Louis, MO, USA).

### 4.2. Conventional Leaching Extraction

Black bean coat was removed manually and ground in a blender (Hamilton beach 80350R). Then it was sieved in a mesh no. 40 before the LC process. A measurement of 40 mg of the sieved coat was extracted with 25 mL of water and ethanol–water (50:50 *v/v*). The solutions were stirred at 150 rpm for 4 h at 40 °C in a stir plate then centrifugated at 2509× *g* for 15 min, and the supernatant was recovered and concentrated in a Büchi rotary evaporator at 60 °C with 180 mbar of pressure. The concentrated extract was stored at −20 °C until further use [13].

### 4.3. Supercritical CO_2_ Fluids Extraction

SFE was performed in a Thar SFE500 extractor (Thar Process, Pittsburgh, PA, USA) equipped with stainless steel extraction cells of 500 mL. A total of 50 g of whole black beans were mixed with 20 g of glass beads in the extraction cell. The carbon dioxide was pressurized and introduced at a 10 g/min flow with 10% water or ethanol–water (50:50 *v/v*) as cosolvents. The equipment was set at 300 Bar and 60 °C, and then the extract was concentrated in a Büchi rotary evaporator equipment at 60 °C with 180 mbar of pressure. The concentrated extract was stored at −20 °C until further use [13].

### 4.4. Determination of Total Phenolic Compounds

Polyphenols were measured using Folin–Ciocalteu´s method. The samples were diluted to a factor of 1:20 with deionized water. A total of 50 µL of the samples, standard or blank (deionized water), were placed in a 96-well plate and then 50 µL of 1 N Folin–Ciocalteu phenol reagent was added. After 5 min, 100 µL of 20% Na_2_CO_3_ were added, and the mixture was incubated for 10 min. The absorbance was read at 690 nm in a multiwell plate reader (TECAN infinite pro 200). The results are expressed as mg gallic acid equivalents (GAE) per g of dried weight (DW) [13].

### 4.5. Determination of Total Anthocyanins

Total anthocyanins were determined by the pH differential method (AOAC Official Method 2005.02). The extracts and samples were diluted to a factor of 1:10 *v/v* with a potassium chloride buffer (pH 1.0, 0.025 M) and sodium acetate (pH 4.5 0.4 M). A total of 200 µL of samples diluted with each pH buffer were transferred to a 96-well microplate reader. Absorbance was read at 520 nm and 700 nm in a multiwell plate reader. The results are expressed as mg of cyanidin-3-glucoside equivalents per gram of bean coat (mg C3GE/g coat) [13].

### 4.6. Phenolic Compounds Purification

The extracts were mixed with amberlite XAD-7HP resin and preconditioned with acidified water (0.3% formic acid). Multiple washes with acidified water were made to remove impurities from the samples. The samples with resin were loaded on a chromatography column (30 × 3 cm) and washed with acidified ethanol 70% (0.3% formic acid) to elute the polyphenols from the resin. The polyphenolic solution was concentrated in a rotary evaporator Büchi and stored at −20 °C [37].

### 4.7. Identification of Phenolic Compounds by ESI-QTOF

ESI-MS analysis of phenolic compound by direct infusion was performed on an ESI-QTOF instrument Water Xevo G2-XS QToF quadrupole time-of-flight mass spectrometry, equipped with an electrospray ionization (ESI) interface (Milford, MA, USA). The MS acquisition was operated in positive and negative ion mode. The parameters were set as follows for positive ion mode: capillary voltage: 3.00 kV, cone voltage: 70 kV, temperature: 150 °C and desolvation temperature: 500 °C. For negative ion mode, capillary voltage: 2.5 kV, cone voltage: 40 kV, temperature: 100 °C and desolvation temperature: 250 °C. The infusion flow rate of the sample was 5 µL/min. Data acquisition and analysis were performed using software MassLynx V4.1, Waters Corporation, Milford, MA, USA.

### 4.8. ABTS Assay

The free radical 2,2′-azino-bis(3-ethylbenzothiazoline)-6-sulfonic acid (ABTS) was made by mixing 100 mL of distilled water with 7 mM ABTS and 2.45 mM Na_2_S_2_O_8_, allowing to stand for 16 h at room temperature in darkness. The solution was adjusted with deionized water to an absorbance of 0.7 ± 0.002 at 734 nm. A measured 20 µL of the extract, blank (deionized water) or Trolox standard were placed in a 96-well microplate. Afterward, 180 µL of ABTS was added and read at an absorbance of 734 nm. The results are expressed as IC_50_ in mg/mL [38].

### 4.9. DPPH Assay

For the 2,2-diphenyl-1-picryl-hydrazil (DPPH) stable radical scavenging assay, 20 µL of the sample, blank (deionized water) or Trolox standard were placed in a 96-well microplate. A measured 180 µL of DPPH (2.36 mg DPPH/100 mL of ethanol 80:20, *v/v*) was added and allowed to stand for 30 min before reading at 517 nm. The results are expressed as IC_50_ in mg/mL [13].

### 4.10. Tyrosinase Inhibition Assay

For tyrosinase inhibition assay, 100 µL of 100 mM phosphate buffer (pH 6.8) and 20 µL of 250 U/mL tyrosinase (diluted in phosphate buffer) were pipetted into a 96 well-plate. Afterward, 20 µL of the extract diluted in deionized water, blank (deionized water) or positive control (kojic acid) were added at various concentrations and incubated for 15 min at room temperature. After incubation, 20 µL of 3 mM L-DOPA was added to each sample, and the reaction was incubated for 15 min. Finally, the absorbance was measured at 474 nm [6].

### 4.11. Elastase Inhibition Assay

25 µL of the Tris-HCl buffer (10 mM, pH 8.0), 25 µL of elastase 0.3 U/mL (diluted in the Tris-HCl buffer) were added in a 96 multiwell plate. Afterward, 25 µL of the extract diluted in deionized water, blank (deionized water) or positive control (catechin) were added at various concentrations and incubated for 15 min at room temperature. After incubation, 25 µL N-succinyl-ala-ala-ala-p-nitroanilide (0.25 mg/mL) was added to each sample, and the reaction was incubated for 15 min. Finally, the absorbance was measured at 410 nm [39]. The inhibition percentage for tyrosinase and elastase assay was calculated with the following equation:

% = ([Abs negative control − (Abs positive control-Abs blank)])/(Abs negative control)(1)

### 4.12. Molecular Docking (In Silico Analysis)

The interaction of phenolic compounds identified by ESI-MS with the enzymes tyrosinase and elastase was evaluated by in silico analysis through molecular docking. Phenolic compounds 3D structures were downloaded from PubChem (www.pubchem.ncbi.nlm.nih.gov) (accessed on 4 August 2021). The 3D structures of mushroom tyrosinase (PDB ID: 2Y9X) and elastase from porcine pancreas (PDB ID: 1BRU) were downloaded from RSCB Protein Data Bank (www.rcsb.org) (accessed on 4 August 2021). Discovery Studio^®^ 2020 was used to clean the structure of the enzyme from water molecules and ligands. Flexible torsions, charges and grid size were assigned using Autodock Tools. Docking calculations were performed using Autodock Vina and the binding pose with the lowest binding energy was selected to be visualized using Discovery Studio visualizer software [40].

### 4.13. Statistical Analysis

The assays were run in triplicate and performed in independent replicates. The data obtained were analyzed using one-way ANOVA by StatPoint STATGRAPHICS Centurion XVI 16.1.03 statistical software (StatPoint Technologies, Inc., Warrenton, VA, USA). Statistical differences among independent variables were determined using Tukey´s Posthoc Test (*p* < 0.05). The IC_50_ values were calculated using GraphPad Prism software 8.0 (GraphPad Software, Inc., San Diego, CA, USA).

## 5. Conclusions

Purified endemic black bean extracts could be used as a cosmeceutical ingredient due to their antioxidant potential and inhibitory potential against tyrosinase and elastase enzymes. LC-purified extract presented higher inhibitory activity in comparison to SFE purified extract. In conventional leaching, black bean seed coats are removed from cotyledons before the process. The cotyledon contains proteins and complex carbohydrates that could be used to develop functional ingredients. According to the ESI-MS analysis, the LC crude extract may contain more phenolic compounds compared to SFE crude extract and the purified extracts. However, it is important to use other analytical methods to confirm the presence of these compounds. Using an adsorption resin for purification allowed the obtention of a concentrated extract free of impurities and toxic solvents. However, it is important to evaluate specific adsorption resins to reduce the phenolic compound loss during the process. On the other hand, specific phenolic compounds such as rutin and catechin could enhance the inhibitory potential against tyrosinase and elastase, respectively. Further cell culture studies are needed to evaluate the reduction in the expression of proteases such as collagenase and elastase in fibroblasts and reduce the process of hyperpigmentation derived from UVR in melanocytes. Endemic black beans (*Phaseolus vulgaris* L.) from Chiapas could be used as a source of phenolic compounds to formulate cosmeceutical products.

## 6. Patents

Title: Extracción y purificación de compuestos fenólicos de frijol común (*Phaseolus vulgaris* L.) con potencial antioxidante y antienvejecimiento; file: MX/a/2020/012916; request ID: 54686.

## Figures and Tables

**Table 1:** Total phenolic compounds and anthocyanins extracts obtained by SFE and leaching process.

Extraction Method	Cosolvent	Total Phenolic Compounds (mg GAE/g Coat)	Anthocyanins (mg C3GE/g Coat)
SFE	H_2_O-100%	63.77 ± 3.16 ^a^	6.76 ± 0.37 ^a^
SFE	H_2_O-EtOH 50%	66.60 ± 7.41 ^a^	7.30 ± 0.64 ^a^
Leaching	H_2_O-100%	44.04 ±1.39 ^b^	3.50 ± 0.35 ^b^
Leaching	H_2_O-EtOH 50%	59.83 ± 4.86 ^a^	5.87 ± 0.21 ^c^

SFE: supercritical fluid extraction, EtOH: ethanol, H_2_O: water, C3GE: Cyanidin 3-glucoside equivalents, GAE: gallic acid equivalents. Different letters indicate significant differences within a column at *p* < 0.05 (Tukey HSD post hoc analysis).

**Table 2:** Tentative phenolic compounds identified by ESI-QTOF using direct infusion analysis.

Sample	Tentative Identification	Elemental Formula Compound	Ion	*m/z* Experimental	*m/z* Theoretical	Tentative Error ppm
Leaching Crude Extract	Quercetin-3-D-Galactoside	C_21_H_20_O_12_	[M-H]−	463.1211	463.1211 *	0
	Malvidin-3-Glucoside	C_23_H_25_O_12_	[M+H]+	331.0715	331.0641 *	−22.3
	Delphinidin 3-Glucoside	C_21_H_20_O_12_	[M+H]+	303.0402	303.0402 *	0
	Cyanidin 3-Glucoside	C_21_H_21_O_11_^+^	[M-H]−	447.1285	447.1242 *	−9.61
	Petunidin-3-O-β-Glucoside	C_22_H_23_O_12_	[M-H]−	447.1285	447.1033	-
	Gallic acid	C_7_H_6_O_5_	[M-H]−	169.0686	169.0606	-
	Sinapic acid	C_11_H_12_O_5_	[M-H]−	223.0993	223.0607	-
	Genistein	C_15_H_10_O_5_	[M-H]−	269.021	269.0455	-
	Protocatechuic acid	C_7_H_6_O_4_	[M-H]−	153.0649	153.0188	-
	Rutin	C_27_H_30_O_16_	[M-H]−	609.1525	609.1461	-
	Naringenin	C_15_H_12_O_5_	[M-H]−	271.0737	271.0612	-
	Catechin	C_15_H_14_O_6_	[M-H]−	289.1219	289.0712	-
	Glycitein	C_16_H_12_O_5_	[M+H]+	285.0356	285.0749	-
	Myricetin	C_15_H_10_O_8_	[M-H]−	317.0705	317.0303	-
	Ferulic acid	C_10_H_10_O_4_	[M-H]−	193.0635	193.0506	-
	Daidzin	C_21_H_20_O_9_	[M-H]−	415.09	415.1	-
	p-coumaric acid	C_9_H_8_O_3_	[M-H]−	163.1649	163.0395	-
	Caffeic acid	C_15_H_10_O_4_	[M-H]−	179.1075	179.0345	-
	Rosmarinic acid	C_18_H_16_O_8_	[M-H]−	359.1975	359.0767	-
Leaching Pure Extract	Quercetin-3-D-Galactoside	C_21_H_20_O_12_	[M-H]−	463.1255	463.1211 *	−9.5
	Malvidin-3-Glucoside	C_23_H_25_O_12_	[M+H]+	331.0715	331.0641 *	−22.3
	Delphinidin 3-Glucoside	C_21_H_20_O_12_	[M+H]+	303.0438	303.0402 *	−11.8
	Cyanidin 3-Glucoside	C_21_H_21_O_11_^+^	[M-H]−	447.1328	447.1242 *	−9.61
	Petunidin-3-O- β -Glucoside	C_22_H_23_O_12_	[M-H]−	447.1422	447.1033	-
	Gallic acid	C_7_H_6_O_5_	[M-H]−	169.0713	169.0606	-
	Protocatechuic acid	C_7_H_6_O_4_	[M-H]−	153.07	153.0188	-
	Rutin	C_27_H_30_O_16_	[M-H]−	609.1423	609.1461	-
	Naringenin	C_15_H_12_O_5_	[M-H]−	271.0737	271.0612	-
	Rosmarinic acid	C_18_H_16_O_8_	[M-H]−	359.2091	359.0767	-
	Catechin	C_15_H_14_O_6_	[M-H]−	289.1184	289.0712	-
	Glycitein	C_16_H_12_O_5_	[M+H]+	285.0356	285.0749	-
	Myricetin	C_15_H_10_O_8_	[M-H]−	317.0823	317.0303	-
	Ferulic acid	C_10_H_10_O_4_	[M-H]−	193.1005	193.0506	-
SFE Crude Extract	Quercetin-3-D-Galactoside	C_21_H_20_O_12_	[M-H]−	463.0462	463.0876 *	89.4
	Cyanidin 3-Glucoside	C_21_H_21_O_11_^+^	[M-H]−	447.0592	447.1242 *	145.3
	Gallic acid	C_7_H_6_O_5_	[M-H]−	169.0207	169.0606	-
	Caffeic acid	C_15_H_10_O_4_	[M-H]−	179.0829	179.0345	-
	Daidzin	C_21_H_20_O_9_	[M-H]−	415.0513	415.1	-
	Sinapic acid	C_11_H_12_O_5_	[M-H]−	223.0014	223.0607	-
	Naringenin	C_15_H_12_O_5_	[M-H]−	271.0333	271.0612	-
	Rosmarinic acid	C_18_H_16_O_8_	[M-H]−	359.1432	359.0767	-
	Catechin	C_15_H_14_O_6_	[M-H]−	289.0766	289.0712	-
	Myricetin	C_15_H_10_O_8_	[M-H]−	317.0568	317.0303	-
	Ferulic acid	C_10_H_10_O_4_	[M-H]−	193.0436	193.0506	-
SFE Pure Extract	Quercetin-3-D-Galactoside	C_21_H_20_O_12_	[M-H]−	463.0462	463.0872 *	89.4
	Cyanidin 3-Glucoside	C_21_H_21_O_11_^+^	[M-H]−	447.0679	447.1242 *	125.9
	Gallic acid	C_7_H_6_O_5_	[M-H]−	169.0367	169.0606	-
	Caffeic acid	C_15_H_10_O_4_	[M-H]−	179.0801	179.0345	-
	Daidzin	C_21_H_20_O_9_	[M-H]−	415.06	415.1	-
	Sinapic acid	C_11_H_12_O_5_	[M-H]−	223.0443	223.0607	-
	Naringenin	C_15_H_12_O_5_	[M-H]−	271.0333	271.0612	-
	Rosmarinic acid	C_18_H_16_O_8_	[M-H]−	359.1432	359.0767	-
	Catechin	C_15_H_14_O_6_	[M-H]−	289.0662	289.0712	-
	Myricetin	C_15_H_10_O_8_	[M-H]−	317.0239	317.0303	-
	Ferulic acid	C_10_H_10_O_4_	[M-H]−	193.0721	193.0506	-

SFE: Supercritical fluid extraction, * symbol indicates the theoretical *m/z* obtained with pure standards, while theoretical *m/z* without the symbol was obtained from MoNa-Mass Bank of North America (https://mona.fiehnlab.ucdavis.edu/) (accessed on 7 July 2021), tentative error ppm was calculated only for the compounds identified with pure standards.

**Table 3:** Molecular docking analysis.

Phenolic Compounds Identified	Predicted Binding Affinity	
	Tyrosinase (kcal/mol)	Elastase (kcal/mol)
Quercetin-3-D-Galactoside	−7.6	−4.8
Malvidin-3-Glucoside	−7.8	−5.5
Delphinidin-3-Glucoside	−7.1	−5.8
Cyanidin-3-Glucoside	−7.7	−3.4
Petunidin-3-O-β-Glucoside	−7.7	−5.7
Gallic acid	−5.9	−5.7
Sinapic acid	−5.9	−4.7
Genistein	−6.9	−5.6
Protocatechuic acid	−5.8	−5.3
Rutin	−8.5	−2.4
Naringenin	−6.8	−6.7
Catechin	−6.8	−6.9
Glycetin	−7.0	−5.1
Myricetin	−6.9	−6.5
Ferulic acid	−5.3	−5.2
Daidzin	−6.6	−4.2
p-coumaric acid	−5.5	−5.0
Caffeic acid	−5.7	−5.2
Rosmarinic acid	−5.9	−6.8
Kojic acid	−5.5	-

## Data Availability

Data from the present study is available upon request.

## References

[1] Kim D.U., Chung H.C., Kim C., Hwang J.K. (2017). Oral Intake of Boesenbergia Pandurata Extract Improves Skin Hydration, Gloss, and Wrinkling: A Randomized, Double-Blind, and Placebo-Controlled Study. J. Cosmet. Dermatol..

[2] Xiong Z.-M., O’Donovan M., Sun L., Choi J.Y., Ren M., Cao K. (2017). Anti-Aging Potentials of Methylene Blue for Human Skin Longevity. Sci. Rep..

[3] Cho Y.H., Bahuguna A., Kim H.H., Kim D., Kim H.J., Yu J.M., Jung H.G., Jang J.Y., Kwak J.H., Park G.H. (2017). Potential Effect of Compounds Isolated from Coffea Arabica against UV-B Induced Skin Damage by Protecting Fibroblast Cells. J. Photochem. Photobiol. B Biol..

[4] Hernandez D.F., Cervantes E.L., Diego A., Luna-Vital L.M. (2020). Food-Derived Bioactive Compounds with Anti-Aging Potential for Nutricosmetic and Cosmeceutical Products. Crit. Rev. Food Sci. Nutr..

[5] Wittenauer J., MäcKle S., Sußmann D., Schweiggert-Weisz U., Carle R. (2015). Inhibitory Effects of Polyphenols from Grape Pomace Extract on Collagenase and Elastase Activity. Fitoterapia.

[6] Şöhretoğlu D., Sari S., Barut B., Özel A. (2018). Tyrosinase Inhibition by Some Flavonoids: Inhibitory Activity, Mechanism by in Vitro and in Silico Studies. Bioorg. Chem..

[7] Chiocchio I., Mandrone M., Sanna C., Maxia A., Tacchini M., Poli F. (2018). Screening of a Hundred Plant Extracts as Tyrosinase and Elastase Inhibitors, Two Enzymatic Targets of Cosmetic Interest. Ind. Crops Prod..

[8] Quideau S., Deffieux D., Douat-casassus C., Pouysegu L. (2011). Natural Products Plant Polyphenols: Chemical Properties, Biological Activities, and Synthesis. Angew. Chem..

[9] Mojica L., Meyer A., Berhow M.A., de Mejía E.G. (2015). Bean Cultivars (Phaseolus Vulgaris L.) Have Similar High Antioxidant Capacity, in Vitro Inhibition of α-Amylase and α-Glucosidase While Diverse Phenolic Composition and Concentration. Food Res. Int..

[10] Hu S., Zhang X., Chen F., Wang M. (2017). Dietary Polyphenols as Photoprotective Agents against UV Radiation. J. Funct. Foods.

[11] Molino A., Mehariya S., Di G., Larocca V., Martino M., Paolo G., Marino T., Chianese S., Balducchi R., Musmarra D. (2020). Recent Developments in Supercritical Fluid Extraction of Bioactive Compounds from Microalgae: Role of Key Parameters, Technological Achievements and Challenges. J. CO2 Util..

[12] Garcia-Vaquero M., Rajauria G., Tiwari B. (2020). Conventional Extraction Techniques: Solvent Extraction.

[13] Hsieh-Lo M., Castillo-Herrera G., Mojica L. (2020). Black Bean Anthocyanin-Rich Extract from Supercritical and Pressurized Extraction Increased In Vitro Antidiabetic Potential, While Having Similar Storage Stability. Foods.

[14] del Garcia-Mendoza M.P., Espinosa-Pardo F.A., Baseggio A.M., Barbero G.F., Maróstica Junior M.R., Rostagno M.A., Martínez J. (2017). Extraction of Phenolic Compounds and Anthocyanins from Juçara (Euterpe Edulis Mart.) Residues Using Pressurized Liquids and Supercritical Fluids. J. Supercrit. Fluids.

[15] Alcázar-Valle M., Lugo-Cervantes E., Mojica L., Morales-Hernández N., Reyes-Ramírez H., Enríquez-Vara J.N., García-Morales S. (2020). Bioactive Compounds, Antioxidant Activity, and Antinutritional Content of Legumes: A Comparison between Four Phaseolus Species. Molecules.

[16] Alfaro-Diaz A., Urías-Silvas J.E., Loarca-Piña G., Gaytan-Martínez M., Prado-Ramirez R., Mojica L. (2021). Techno-Functional Properties of Thermally Treated Black Bean Protein Concentrate Generated through Ultrafiltration Process. LWT.

[17] Escobedo A., Loarca-Piña G., Gaytan-Martínez M., Orozco-Avila I., Mojica L. (2020). Autoclaving and Extrusion Improve the Functional Properties and Chemical Composition of Black Bean Carbohydrate Extracts. J. Food Sci..

[18] Gross J.H. (2014). Direct Analysis in Real Time-a Critical Review on DART-MS. Anal. Bioanal. Chem..

[19] Carbas B., Machado N., Oppolzer D., Ferreira L., Queiroz M., Brites C., Rosa E.A.S., Barros A.I.R.N.A. (2020). Nutrients, Antinutrients, Phenolic Composition, and Antioxidant Activity of Common Bean Cultivars and Their Potential for Food Applications. Antioxidants.

[20] Pratheeshkumar P., Son Y.O., Wang X., Divya S.P., Joseph B., Hitron J.A., Wang L., Kim D., Yin Y., Roy R.V. (2014). Cyanidin-3-Glucoside Inhibits UVB-Induced Oxidative Damage and Inflammation by Regulating MAP Kinase and NF-ΚB Signaling Pathways in SKH-1 Hairless Mice Skin. Toxicol. Appl. Pharmacol..

[21] Luthria D.L., Pastor-Corrales M.A. (2006). Phenolic Acids Content of Fifteen Dry Edible Bean (*Phaseolus vulgaris* L.) Varieties. J. Food Compos. Anal..

[22] Mojica L., Berhow M., Gonzalez de Mejia E. (2017). Black Bean Anthocyanin-Rich Extracts as Food Colorants: Physicochemical Stability and Antidiabetes Potential. Food Chem..

[23] Yang Q., Gan R., Ge Y., Zhang D., Corke H. (2018). Polyphenols in Common Beans (*Phaseolus vulgaris* L.): Chemistry, Analysis, and Factors Affecting Composition. Compr. Rev. Food Sci. Food Saf..

[24] Jiao X., Zhang X., Zhang Q., Gao N., Li B., Meng X. (2017). Optimation of Enrichment and Purification of Polyphenols from Blueberries (*Vaccinium* Spp.) by Macroporous Resins XAD-7HP. Emirates J. Food Agric..

[25] Mat Saad H., Tan C.H., Lim S.H., Manickam S., Sim K.S. (2021). Evaluation of Anti-Melanogenesis and Free Radical Scavenging Activities of Five Artocarpus Species for Cosmeceutical Applications. Ind. Crops Prod..

[26] Rittié L., Fisher G.J. (2015). Natural and Sun-Induced Aging of Human Skin. Cold Spring Harb. Perspect. Med..

[27] Jabłońska-Trypuć A., Matejczyk M., Rosochacki S. (2016). Matrix Metalloproteinases (MMPs), the Main Extracellular Matrix (ECM) Enzymes in Collagen Degradation, as a Target for Anticancer Drugs. J. Enzym. Inhib. Med. Chem..

[28] Kusumawati I., Indrayanto G. (2013). Natural Antioxidants in Cosmetics.

[29] Burger P., Landreau A., Azoulay S., Michel T., Fernandez X. (2016). Skin Whitening Cosmetics: Feedback and Challenges in the Development of Natural Skin Lighteners. Cosmetics.

[30] Mukherjee P.K., Biswas R., Sharma A., Banerjee S., Biswas S., Katiyar C.K. (2018). Validation of Medicinal Herbs for Anti-Tyrosinase Potential. J. Herb. Med..

[31] Ersoy E., Eroglu Ozkan E., Boga M., Yilmaz M.A., Mat A. (2019). Anti-Aging Potential and Anti-Tyrosinase Activity of Three Hypericum Species with Focus on Phytochemical Composition by LC–MS/MS. Ind. Crops Prod..

[32] Athipornchai A., Jullapo N. (2018). Tyrosinase Inhibitory and Antioxidant Activities of Orchid (*Dendrobium* Spp.). S. Afr. J. Bot..

[33] Abdul Karim A., Azlan A., Ismail A., Hashim P., Abd Gani S.S., Zainudin B.H., Abdullah N.A. (2014). Phenolic Composition, Antioxidant, Anti-Wrinkles and Tyrosinase Inhibitory Activities of Cocoa Pod Extract. BMC Complementary Altern. Med..

[34] Wang L., Lee W.W., Cui Y.R., Ahn G., Jeon Y.J. (2019). Protective Effect of Green Tea Catechin against Urban Fine Dust Particle-Induced Skin Aging by Regulation of NF-ΚB, AP-1, and MAPKs Signaling Pathways. Environ. Pollut..

[35] Pientaweeratch S., Panapisal V., Tansirikongkol A. (2016). Antioxidant, Anti-Collagenase and Anti-Elastase Activities of Phyllanthus Emblica, Manilkara Zapota and Silymarin: An in Vitro Comparative Study for Anti-Aging Applications. Pharm. Biol..

[36] Eun Lee K., Bharadwaj S., Yadava U., Gu Kang S. (2019). Evaluation of Caffeine as Inhibitor against Collagenase, Elastase and Tyrosinase Using in Silico and in Vitro Approach. J. Enzym. Inhib. Med. Chem..

[37] Johnson M.H., De Mejia E.G., Fan J., Lila M.A., Yousef G.G. (2013). Anthocyanins and Proanthocyanidins from Blueberry-Blackberry Fermented Beverages Inhibit Markers of Inflammation in Macrophages and Carbohydrate-Utilizing Enzymes in Vitro. Mol. Nutr. Food Res..

[38] Nenadis N., Wang L.F., Tsimidou M., Zhang H.Y. (2004). Estimation of Scavenging Activity of Phenolic Compounds Using the ABTS + Assay. J. Agric. Food Chem..

[39] Rodrigues M.J., Pereira C.A., Oliveira M., Neng N.R., Nogueira J.M.F., Zengin G., Mahomoodally M.F., Custódio L. (2018). Sea Rose (Armeria Pungens (Link) Hoffmanns. & Link) as a Potential Source of Innovative Industrial Products for Anti-Ageing Applications. Ind. Crops Prod..

[40] San Pablo-Osorio B., Mojica L., Urías-Silvas J.E. (2019). Chia Seed (*Salvia hispanica* L.) Pepsin Hydrolysates Inhibit Angiotensin-Converting Enzyme by Interacting with Its Catalytic Site. J. Food Sci..

